# Magnetic metasurfaces properties in the near field regions

**DOI:** 10.1038/s41598-022-07378-y

**Published:** 2022-02-28

**Authors:** Danilo Brizi, Agostino Monorchio

**Affiliations:** 1 Department of Information Engineering, University of Pisa, 56122 Pisa, Italy; 2 Consorzio Nazionale Interuniversitario per le Telecomunicazioni (CNIT), 43124 Parma, Italy

**Keywords:** Electrical and electronic engineering, Electronic and spintronic devices

## Abstract

In this paper, we present a general equivalent-circuit interpretation of finite magnetic metasurfaces interacting with an arbitrary arrangement of RF coils operating in near-field regime. The developed model allows to derive a physical interpretation of the interactions between the metasurface and the surrounding RF coils, both transmitting and receiving. Indeed, especially for near-field applications, the metasurface presence modifies the behavior of each RF coil differently, due to the specific reciprocal interactions. Hence, the proposed approach introduces a source-related complex magnetic permeability matrix, overcoming the traditional bulk definition. To prove the model validity against full-wave simulations, we present two significant test cases, commonly used in practical applications. The former is represented by the simple metasurface-coil arrangement from which important and fundamental considerations can be drawn. The latter system is composed by a transmitting and a receiving coil with a metasurface in between; detailed explanations on the metasurface interactions with both the RF coils are developed. Finally, we also achieved an excellent agreement between the numerical results and the measurements obtained through fabricated prototypes. In summary, the circuit interpretation herein presented, in addition to the rigorous electromagnetic theoretical approaches already appeared in the open literature, reveals useful in providing quantitative, practical, and easy-to-handle guidelines for the design and physical understanding of finite magnetic metasurfaces interacting with arbitrary RF coils arrangements in the near-field regime.

## Introduction

Metamaterials and metasurfaces represent nowadays a consolidated and important branch of the electromagnetic research activity^1,2^. The huge surge of interest towards these structures arises because of their extraordinary properties in terms of dielectric permittivity and magnetic permeability. Indeed, it has been demonstrated that negative permittivity or permeability and even negative refractive index metamaterials can be realized, starting from a proper unit-cell design^3–7^. The common feature of all the metamaterials or metasurfaces is that they are constituted by a periodic 2D or 3D arrangement of resonant unit-cells: the non-natural properties come from the resonant behavior of the single unit-cell^8–10^. By modulating this behavior, the desired working frequency, bandwidth and strength of the dielectric and magnetic response can be established. Another fundamental design requisite for these structures relies in the subwavelength regime for the unit-cells, i.e. the unit-cell must be significantly smaller than the field wavelength. This specification allows the adoption of the homogenization hypothesis^11–14^; therefore, the relatively long wavelength of the impinging electromagnetic field is not able to distinguish the elementary structure of the metamaterial, that can be interpreted as homogeneous.

Among this broad class, magnetic metasurfaces (either isotropic and anisotropic) have been studied for their interesting properties with respect to the impinging magnetic field^1^. Indeed, when such structures are employed in the low-frequency regime (quasi-static magnetic field), electric and magnetic field are decoupled and, thus, only the magnetic permeability control is required to accomplish the performance enhancement needed by a specific application^15,16^. As an example, Magnetic Resonance Imaging (MRI) and resonant inductive Wireless Power Transfer (WPT) are two important communities in which magnetic metasurfaces recently gained a considerable interest^16–22^. Typically, magnetic metasurfaces are in the form of arrays exploiting resonant magnetic inclusions, like spiral resonators, split rings and similar^15,23–28^.

In the literature, rigorous physical interpretations have been provided, based on the classical electromagnetic theory^15,23,29,30^: all the electromagnetic aspects and interactions between the magnetic field and metamaterial are therein described through Maxwell equations and some experimental verifications of the theoretical predictions have been carried out.

Although rigorous and formally complete, these interpretations are often difficult to be employed in order to derive practical design considerations and guidelines; indeed, workable and comprehensive guidelines, that can be particularly useful in every engineering activity to make the design easier and more effective, are still lacking^24–26^. In addition, the theoretical models are also frequently bound to simplifying hypotheses, required to derive closed mathematical relationships. For instance, the coils are generally considered extremely small with respect not only to the wavelength (quasi-static hypothesis) but also to the distance between them and the other elements of the considered set-up^23,30^; moreover, an impinging plane wave hypothesis is typically adopted. However, in practical applications, these conditions are often far from being met, thus the metasurface behavior is different from what theoretically predicted.

The purpose of this paper is to provide general and practical design guidelines based on an equivalent circuit interpretation of finite magnetic metasurfaces interacting with RF coils operating in near-field regime, presenting, at the same time, a physical understanding of the complex magnetic permeability by using the same derived lumped elements. The proposed model is completely general and it can be applied to arbitrary RF coils arrangements, overcoming other existent similar circuital interpretation^31–35^. In particular, for the first time to the best of our knowledge, we propose a source-related complex magnetic permeability, differently from the typical approach where it is retrieved as a bulk property^36^. We show how each RF coil of the considered arrangement experiences a different permeability value, depending on the relative position with respect to the metasurface. Such interpretation reveals especially suitable to describe near-field interactions between the RF coils and the metasurface. All these aspects can greatly simplify the achievement of the metasurface desired behavior, by filling the gap between a pure physical interpretation and real applications.

In the literature, it was demonstrated that a metasurface can be described, in its entireness, by an equivalent *RLC* circuit, by starting from the single unit-cell design and their relative positioning inside the array^37^. However, in that work, no explanation or physical understanding about the interactions between the metasurface and the nearby RF coils was provided. To overcome this limitation, we herein present the analytical model to retrieve the source-related complex magnetic permeability values, by opportunely modifying the impedance matrix of a generic RF coils arrangement. The proposed approach allows to fully understand the interactions between a magnetic metasurface and an arbitrary RF coils arrangement, in a quantitative way. Then, as an example to test the developed model, we present some meaningful and practical set-ups commonly employed in different fields, as for instance in MRI and WPT. Specifically, we focus on the case of one RF coil interacting with a magnetic metasurface at first; secondly, the case of two distinct RF coils simultaneously interacting with a metasurface is introduced. We then compare our model predictions with full-wave simulations, to validate the circuital approach against complete Maxwell equations. Finally, we performed experimental measurements over fabricated prototypes.

The paper is organized as follows; first, the proposed equivalent circuit model is developed; specific emphasis will be directed to analyze the effect of the metasurface on the system RF coils in terms of source-related complex magnetic permeability values. After that, the adopted test-cases CAD models and the corresponding fabricated prototypes are described, together with their equivalent circuit characterization. A specific section is devoted to providing practical design guidelines and physical considerations, exploiting the meaningful test-cases above proposed and reporting the results comparison between the analytical model, full-wave simulations and experimental measurements. Finally, a summarizing discussion follows.

## Results and methods

### Proposed equivalent circuit model

**Figure 1:**
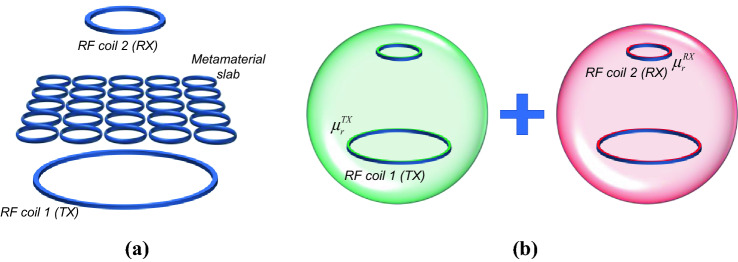
(**a**) Pictorial representation of a typical magnetic metasurface (constituted by a matrix of resonators) with the presence of two generic RF coils. (**b**) The RF coils are immersed in an equivalent medium characterized by the source-related relative permeability $\mu_{r}^{TX,RX}$ due to the metasurface presence.

The typical magnetic metasurface configuration comprises an array of resonant unit-cells, like spiral or split-ring resonators (Fig. 1a). The whole array reacts to an impinging magnetic field with a resonant behavior (usually described through a Lorentzian model), making possible to exploit enhanced and *μ*-negative permeability at specific bandwidths. It has been demonstrated in^37^ that the entire metasurface can be represented by an equivalent *RLC* model; this step is fundamental to describe and quantify its interactions with other RF coils, as it will be shown in the following sections. A brief recall of the metasurface *RLC* reduction is herein reported for the reader clarity: more details can be found in^37^. In a generic arrangement, we can have *M* fed RF coils interacting with a passive metasurface. The metasurface can be assumed to be formed by *N* × *N* = *P* resonant unit-cells. If we refer to the RF coils with the first *M* indices and with the following *P* indices to the elements of the array, the overall system impedance matrix can be written as below.

1 $$\left( {\begin{array}{*{20}c} {Z_{11} } & {...} & {Z_{1M} } & {...} & {Z_{1(P + M)} } \\ \vdots & \vdots & \vdots & \vdots & \vdots \\ {Z_{M1} } & {...} & {Z_{MM} } & {...} & {Z_{M(P + M)} } \\ {Z_{{\left( {M + 1} \right)1}} } & {...} & {Z_{{\left( {M + 1} \right)M}} } & {...} & {Z_{{\left( {M + 1} \right)(P + M)}} } \\ \vdots & \vdots & \vdots & \vdots & \vdots \\ {Z_{{\left( {M + P} \right)1}} } & {...} & {Z_{{\left( {M + P} \right)M}} } & {...} & {Z_{{\left( {M + P} \right)\left( {M + P} \right)}} } \\ \end{array} } \right)\left( {\begin{array}{*{20}c} {I_{1} } \\ \vdots \\ {I_{M} } \\ {c_{{\left( {M + 1} \right)}} I_{x} } \\ \vdots \\ {c_{{\left( {M + P} \right)}} I_{x} } \\ \end{array} } \right) = \left( {\begin{array}{*{20}c} V \\ \vdots \\ {V_{M} } \\ 0 \\ \vdots \\ 0 \\ \end{array} } \right)$$

in which we express the currents flowing in each element of the metasurface in the following form:

2 $$I_{i} = c_{i} I_{x} ,{ }\quad with\;i = M + 1, \ldots ,M + P$$

where *c*_*i*_ is the generic *i*-th complex current coefficient and *I*_*x*_ is the equivalent current flowing in the *RLC* model of the array. By summing up equations from row *M* + 1 to row *M* + *P* and re-arranging terms, it is possible to write the following system, where the *P* elements of the metasurface have been substituted by their equivalent resonator (marked with index *x*).

3 $$\left( {\begin{array}{*{20}c} {Z_{11} } & \cdots & {Z_{1M} } & {Z_{1x} } \\ \vdots & \vdots & \vdots & \vdots \\ {Z_{M1} } & \cdots & {Z_{MM} } & {Z_{Mx} } \\ {Z_{x1} } & \cdots & {Z_{xM} } & {Z_{xx} } \\ \end{array} } \right)\left( {\begin{array}{*{20}c} {I_{1} } \\ \vdots \\ {I_{M} } \\ {I_{x} } \\ \end{array} } \right) = \left( {\begin{array}{*{20}c} {V_{1} } \\ \vdots \\ {V_{M} } \\ 0 \\ \end{array} } \right)$$

In particular, the *Z*_*xx*_ term can be interpreted as the self-impedance of the metasurface equivalent resonator (*RLC* series), whereas *I*_*x*_ is its equivalent flowing current and the various *Z*_*xi*_ terms correspond to the mutual coupling coefficients between the metasurface and each of the *M* RF coils^37^.

At this point, we can express the current *I*_*x*_ that flows in the equivalent metasurface *RLC* circuit as a function of the other *M* RF coils currents, exploiting the equations system (3):

4 $$I_{x} = - \frac{{\sum\nolimits_{i = 1}^{M} {Z_{xi} I_{i} } }}{{Z_{xx} }}$$

Thus, we can substitute the expression (4) in the first *M* equations of (3); therefore, the effect of the metasurface presence over the other *M* RF coils can be easier highlighted:

5 $$\left( {\begin{array}{*{20}c} {Z_{11} - \frac{{Z_{1x} Z_{x1} }}{{Z_{xx} }}} & {Z_{12} - \frac{{Z_{1x} Z_{x2} }}{{Z_{xx} }}} & \cdots & {Z_{1M} - \frac{{Z_{1x} Z_{xM} }}{{Z_{xx} }}} \\ {Z_{21} - \frac{{Z_{2x} Z_{x1} }}{{Z_{xx} }}} & {Z_{22} - \frac{{Z_{2x} Z_{x2} }}{{Z_{xx} }}} & \cdots & {Z_{2M} - \frac{{Z_{2x} Z_{xM} }}{{Z_{xx} }}} \\ \vdots & \vdots & \vdots & \vdots \\ {Z_{M1} - \frac{{Z_{Mx} Z_{x1} }}{{Z_{xx} }}} & \cdots & \cdots & {Z_{MM} - \frac{{Z_{Mx} Z_{xM} }}{{Z_{xx} }}} \\ \end{array} } \right)\left( {\begin{array}{*{20}c} {I_{1} } \\ {I_{2} } \\ \vdots \\ {I_{M} } \\ \end{array} } \right) = \left( {\begin{array}{*{20}c} {V_{1} } \\ {V_{2} } \\ \vdots \\ {V_{M} } \\ \end{array} } \right)$$

Further, we can write the generic element of the impedance matrix in (5) by introducing the source-related complex (relative) magnetic permeability value $\mu_{r}^{ij}$, as described below.

6 $$Z_{ij} \left( {1 - \frac{{Z_{ix} Z_{xj} }}{{Z_{xx} Z_{ij} }}} \right) = \mu_{r}^{ij} Z_{ij} \quad with\;i,j = 1,2, \ldots ,M$$

As it will be better clarified for the adopted test-cases, each RF coil undergoes to a unique impedance modification due to the presence of the magnetic metasurface, in dependence of its relative position and interactions with other elements. Thus, each RF coil, and each corresponding mutual coupling term of the impedance matrix (5), experiences a different equivalent complex permeability value (what we call source-related permeability, Fig. 1b). Finally, the overall *M* RF coils can be represented by the following complete equations system, where the metasurface presence has been translated into the complex (relative) magnetic permeability coefficients $\mu_{r}^{ij}$:

7 $$\left( {\begin{array}{*{20}c} {\mu_{r}^{11} Z_{11} } & {\mu_{r}^{12} Z_{12} } & \cdots & {\mu_{r}^{1M} Z_{1M} } \\ {\mu_{r}^{21} Z_{21} } & {\mu_{r}^{22} Z_{22} } & \cdots & {\mu_{r}^{2M} Z_{2M} } \\ \vdots & \vdots & \vdots & \vdots \\ {\mu_{r}^{M1} Z_{M1} } & \cdots & \cdots & {\mu_{r}^{MM} Z_{MM} } \\ \end{array} } \right)\left( {\begin{array}{*{20}c} {I_{1} } \\ {I_{2} } \\ \vdots \\ {I_{M} } \\ \end{array} } \right) = \left( {\begin{array}{*{20}c} {V_{1} } \\ {V_{2} } \\ \vdots \\ {V_{M} } \\ \end{array} } \right)$$

Therefore, practical guidelines and physical interpretations useful to accomplish the desired design can be derived from the retrieved lumped elements of the entire system, by using the complex relative permeability matrix $\underline{\underline{{\mu_{r} }}}$. Indeed, additional degrees of freedom are available to the designer to optimize the *M* RF coils system, consequently exploiting more effectively its potentialities through the introduced source-related magnetic permeability values.

### Selected experimental set-ups

It is worth remarking that the aim of this paper is to develop a circuit-based model able to provide useful and practical design guidelines for the realization of a finite magnetic metasurface interacting with a generic RF coils arrangement, also giving a physical interpretation of the entire system by using the retrieved complex magnetic permeability matrix, as previously explained. Therefore, we herein report two meaningful test-cases adopted to validate the proposed approach. Firstly, a single RF coil-metasurface system is faced; this simple configuration can be seen as the building block of several applications, as for instance in Magnetic Resonance Imaging RF coil design^38^. Secondly, the system formed by a transmitting coil, a metasurface and a receiving coil is analyzed with our circuit model (as schematically depicted in Fig. 1a): for this case, some important and effective design considerations can be drawn, especially suitable for resonant inductive Wireless Power Transfer applications^19^. Nonetheless, the provided analysis is completely general and can be applied also to more complex coils arrangements.

Specifically, we exploited a Method of Moments electromagnetic solver (Feko suite, Altair, Troy, MI, USA) for the entire design process while the measurements have been performed by using the Keysight (Santa Rosa, CA, USA) N9918B FieldFox Handheld Vector Network Analyzer.

#### Single coil-metasurface system description

**Figure 2:**
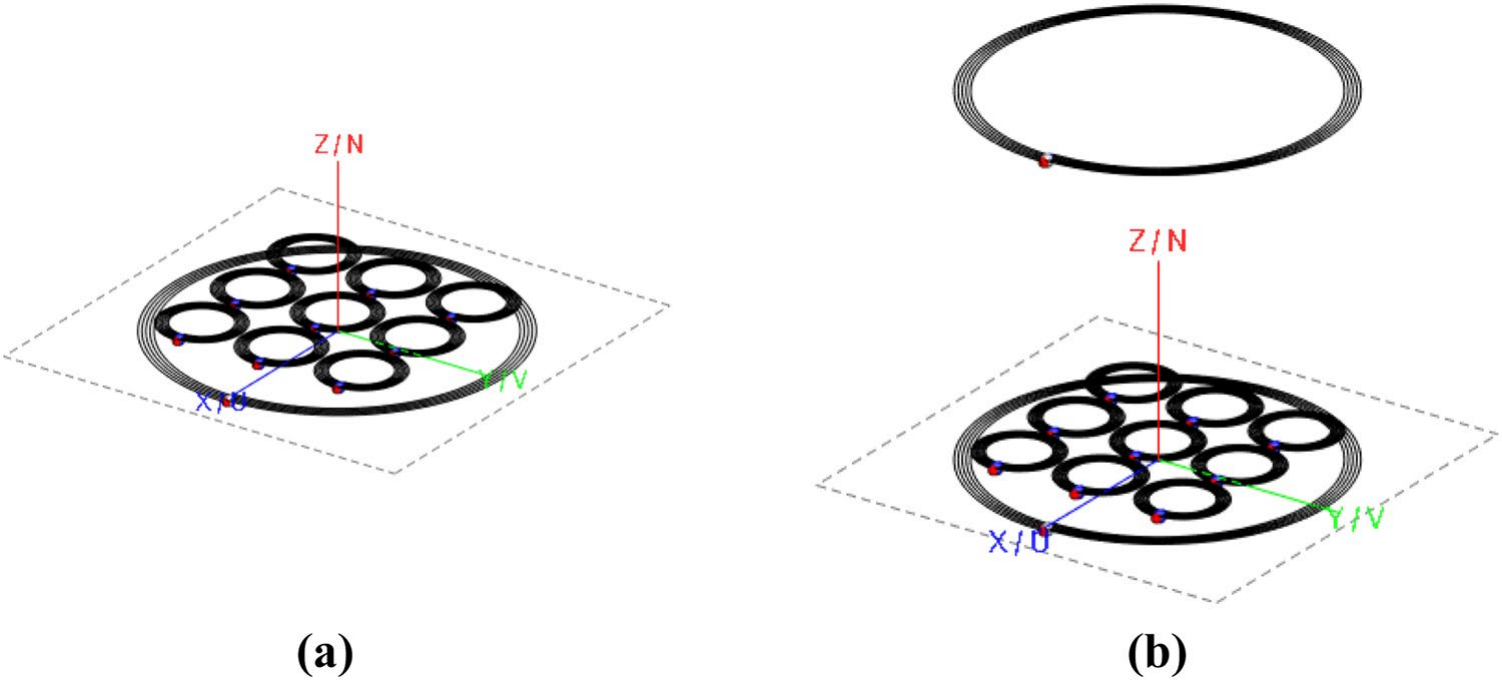
Test-cases CAD models: single coil-metasurface set-up (**a**), transmitter-metasurface-receiver configuration (**b**).

The first proposed test-case is depicted in Fig. 2a. It comprises an RF active planar spiral with a 10 cm external diameter. The coil presents 5 turns of a 28 AWG lossy copper wire, with a pitch between adjacent branches of 0.68 mm. No additional reactive loads are added, and the spiral is non-resonant.

We also consider a metasurface made of a planar 3 × 3 structure; each unit-cell is an 8-turns passive resonant spiral with a 2.4 cm external diameter. The unit-cell pitch is 0.18 mm, made of 28 AWG lossy copper wire. The overall metasurface is positioned 5 mm away from the active RF coil, in a coaxial fashion. In order to operate at the desired working frequency (around 6 MHz), a 390 pF capacitor is added in series to each unit-cell. The choice of the working frequency is arbitrary and other values might have been chosen as well. Following the methodology reported in^37^, we extracted the equivalent *RLC* model of the metasurface together with the mutual coupling coefficient with the active RF coil. The obtained values are: *R*_*meta*_ = 4.13 Ω, *L*_*meta*_ = 14.97 μH, *C*_*meta*_ = 43.35 pF, *M*_*meta-coil*_ = 2.09 μH.

**Figure 3:**
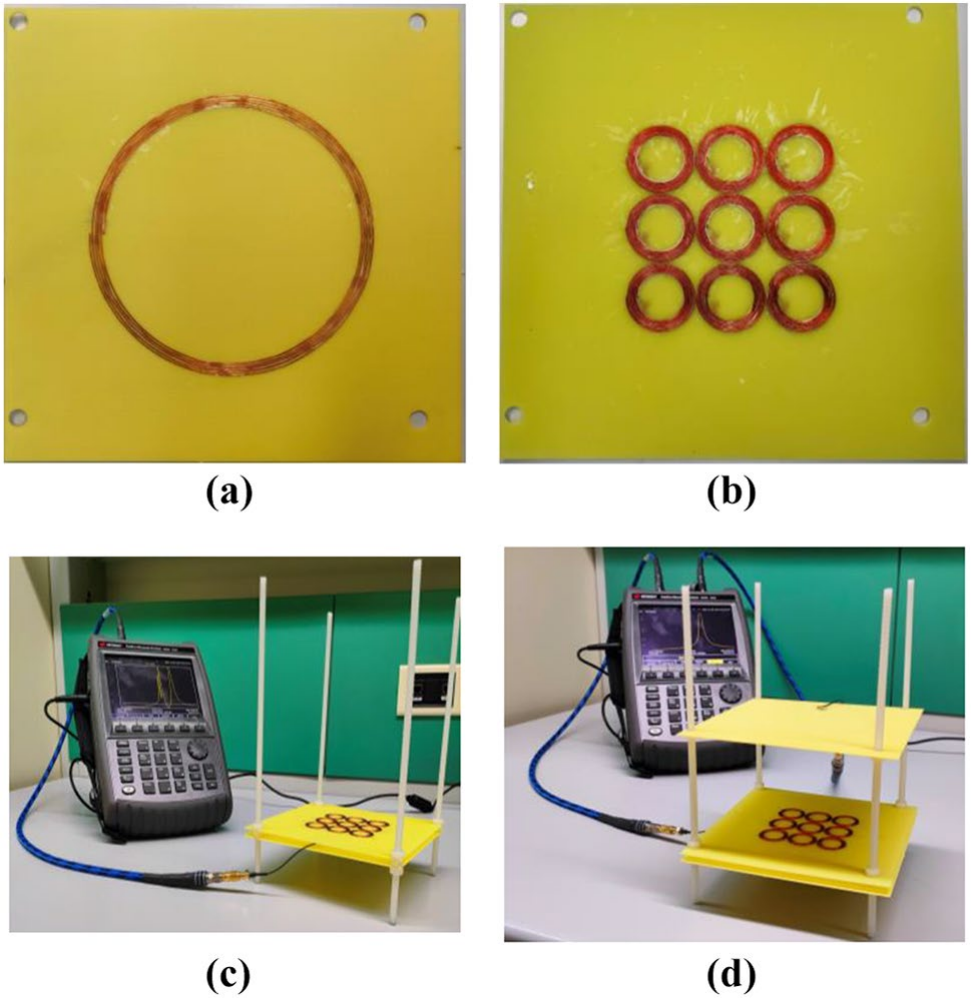
Fabricated prototypes: transmitter/receiver coil (**a**), proposed metasurface (**b**). In (**c**) and (**d**), the complete experimental set-ups are reported for the two configurations.

Besides the numerical simulations, we also fabricated prototypes to perform experimental measurements (Fig. 3a, b). The prototypes are built with a 28 AWG copper wire glued onto an 0.8 mm thick FR4 slab (ε_r_ = 4.3, tanδ = 0.02). The capacitors are soldered on the other side, following the design specifications. In addition, Fig. 3c shows the final experimental arrangement, where a plastic framework is employed to precisely positioning the radiating elements in terms of distances, exploiting the 4 external holes drilled on the FR4 substrate.

#### Transmitter-metasurface-receiver system description

The second CAD configuration is shown in Fig. 2b. Essentially, it consists in the same configuration of the previous test-case but an additional RF passive coil is added. This coil is geometrically identical to the fed RF spiral and it is non-resonant (thus, it is not loaded with capacitors). It has been placed 10 cm away from the fed one, always in a coaxial fashion. This arrangement is typically used in inductive WPT, where a transmitting coil, a metasurface and a receiving coil are positioned as in this example.

As in the previous case, we also extracted the mutual coupling coefficient between the metasurface and the added receiving coil. The other lumped values, i.e. the metasurface equivalent *RLC* and its mutual coupling with the fed coil, have been already calculated from the first configuration. The coefficient *M*_*meta-receiver*_ was estimated equal to 0.12 μH.

Finally, also for this test-case, the experimental set-up was arranged (Fig. 3d).

### Physical interpretation and practical design guidelines

#### Single coil-metasurface system

Once we model a magnetic metasurface through its own equivalent circuit, then we can write the equations that rule the case under consideration:

8 $$\left\{ {\begin{array}{*{20}c} {Z_{11} I_{1} + Z_{12} I_{2} = V_{1} } \\ {Z_{21} I_{1} + Z_{22} I_{2} = 0} \\ \end{array} } \right.$$

where the RF coil is indicated with the index 1, whereas the metasurface is globally reduced to its single equivalent resonator and pointed out with index 2. By expressing the current *I*_2_ as a function of *I*_1_, it is straightforward writing down the impedance seen at the port 1:

9 $$Z_{in} = Z_{11} - \frac{{Z_{12} Z_{21} }}{{Z_{22} }}$$

We can now exploit the developed analytical model to elaborate equation (9); in particular, we assume that the RF coil (element 1) is not loaded with any capacitor, thus it is represented by its self-resistance and inductance:

10 $$Z_{in} = R_{1} + j\omega L_{1} + \frac{{\omega^{2} M_{12}^{2} }}{{R_{2} + j\omega L_{2} + {\raise0.7ex\hbox{$1$} \!\mathord{\left/ {\vphantom {1 {j\omega C_{2} }}}\right.\kern-\nulldelimiterspace} \!\lower0.7ex\hbox{${j\omega C_{2} }$}}}}$$

Through some algebraic manipulations, we can express the port impedance in the following form:

11 $$Z_{in} = R_{1} + j\omega L_{1} \left[ {1 + \frac{{\omega^{2} M_{12}^{2} {\raise0.7ex\hbox{${L_{1} }$} \!\mathord{\left/ {\vphantom {{L_{1} } {C_{2} }}}\right.\kern-\nulldelimiterspace} \!\lower0.7ex\hbox{${C_{2} }$}} - \omega^{4} M_{12}^{2} L_{1} L_{2} }}{{\omega^{2} L_{1}^{2} R_{2}^{2} + \left( {{\raise0.7ex\hbox{${L_{1} }$} \!\mathord{\left/ {\vphantom {{L_{1} } {C_{2} }}}\right.\kern-\nulldelimiterspace} \!\lower0.7ex\hbox{${C_{2} }$}} - \omega^{2} L_{1} L_{2} } \right)^{2} }}} \right.\left. { - j\frac{{\omega^{3} M_{12}^{2} L_{1} R_{2} }}{{\omega^{2} L_{1}^{2} R_{2}^{2} + \left( {{\raise0.7ex\hbox{${L_{1} }$} \!\mathord{\left/ {\vphantom {{L_{1} } {C_{2} }}}\right.\kern-\nulldelimiterspace} \!\lower0.7ex\hbox{${C_{2} }$}} - \omega^{2} L_{1} L_{2} } \right)^{2} }}} \right]$$

At this point, we can introduce the source-related complex (relative) magnetic permeability *μ*_*r*_; this permeability is associated to the equivalent medium in which the RF coil 1 is immersed (Fig. 1b):

12 $$Z_{in} = R_{1} + j\omega (\mu_{r}^{{\prime }} - j\mu_{r}^{{\prime \prime }} )L_{1}$$

and, thus, we can express this equivalent complex relative permeability as a function of the lumped elements of our circuit equivalent model:

13 $$\left\{ {\begin{array}{*{20}c} {\mu_{r}^{{\prime }} = 1 + \frac{{\omega^{2} M_{12}^{2} {\raise0.7ex\hbox{${L_{1} }$} \!\mathord{\left/ {\vphantom {{L_{1} } {C_{2} }}}\right.\kern-\nulldelimiterspace} \!\lower0.7ex\hbox{${C_{2} }$}} - \omega^{4} M_{12}^{2} L_{1} L_{2} }}{{\omega^{2} L_{1}^{2} R_{2}^{2} + \left( {{\raise0.7ex\hbox{${L_{1} }$} \!\mathord{\left/ {\vphantom {{L_{1} } {C_{2} }}}\right.\kern-\nulldelimiterspace} \!\lower0.7ex\hbox{${C_{2} }$}} - \omega^{2} L_{1} L_{2} } \right)^{2} }}} \\ {\mu_{r}^{{\prime \prime }} = \frac{{\omega^{3} M_{12}^{2} L_{1} R_{2} }}{{\omega^{2} L_{1}^{2} R_{2}^{2} + \left( {{\raise0.7ex\hbox{${L_{1} }$} \!\mathord{\left/ {\vphantom {{L_{1} } {C_{2} }}}\right.\kern-\nulldelimiterspace} \!\lower0.7ex\hbox{${C_{2} }$}} - \omega^{2} L_{1} L_{2} } \right)^{2} }}} \\ \end{array} } \right.$$

**Figure 4:**
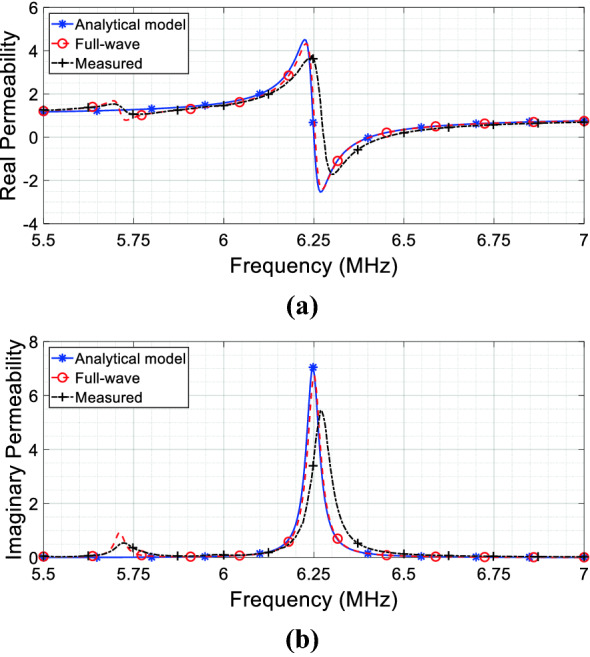
Complex relative magnetic permeability calculated from (13) using the extracted lumped values versus full-wave and measured retrieved permeability: (**a**) real component; (**b**) imaginary component.

In order to report the complex magnetic permeability behavior versus frequency expressed by the Eq. (13), we used the lumped elements values retrieved from the CAD model described in Fig. 2a; the results are shown in Fig. 4a ($\mu_{r}^{{\prime }}$) and Fig. 4(b) ($\mu_{r}^{{\prime \prime }}$). In these graphs, we also compared the pure analytically retrieved permeability against full-wave simulations and experimental measurements. As evident from Fig. 4, we observe an excellent agreement, thus demonstrating the reliability of the circuit model. It may be worth highlighting that this is the equivalent magnetic permeability as seen by the RF coil 1 itself; thus, it does not represent the actual bulk permeability of the metasurface alone. Hence, differently from the canonical approach, we avoid describing the bulk permeability of the proposed metasurface; instead, the metasurface equivalent effect onto the medium surrounding the RF coils arrangement is pointed out (from which the term source-related permeability).

**Figure 5:**
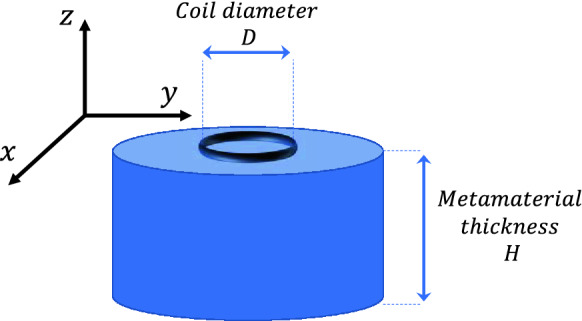
Pictorial representation of an RF coil placed in the close proximity of a thick metamaterial (*H* > *D*); above the coil, it is supposed to be air.

**Figure 6:**
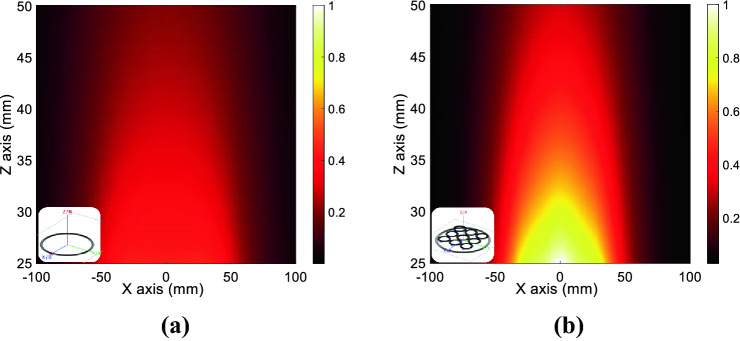
Numerical magnetic field maps evaluated for the configuration shown in the inset on a plane perpendicular to the metasurface (*xz* plane in Fig. 2a): actively fed RF coil without (**a**) and with (**b**) metasurface, in the condition of the same circulating current. As evident, the metasurface presence with a *μ*_*r*_ =  − 1 behavior is able to significantly enhance the magnetic field amplitude, in according to the theoretical model.

In this sense, a noticeable result that has been proved in the literature is that a *μ*_*r*_ = −1 metamaterial can enhance the evanescent magnetic field produced by an RF coil^15,30,38^. Hence, the question is how a metamaterial with a *μ*_*r*_ = −1 as its own bulk permeability interacts with the RF coil from a circuital point of view. As typically presented in the literature, a metamaterial can be simulated by a numerical solver and it consists of a thick slab of homogeneous material showing the desired permeability. As a matter of fact, the slab thickness is often larger than the diameter of the RF coil placed in its proximity^30,38^ (see Fig. 5). In addition, it is also positioned very close to the coil. Since the electromagnetic field produced by a resonator significantly drops for distances larger than its diameter^7^, this configuration corresponds to divide the space where the RF coil is placed into two subdomains: a homogenous material with *μ*_*r*_ = −1 at one side and free space on the other side (*μ*_*r*_ = +1) (Fig. 5). Thus, it is reasonable to expect that the effective magnetic permeability seen by the RF coil will be the average value of the permeability of the two subdomains; this implies that the equivalent medium permeability is zero in its real component (see (13)). Therefore, according to (12), this condition has the effect of cancelling the reactive component of the RF coil impedance, thus putting the coil under resonance. By referring to the results of Fig. 4, the zero value for permeability happens at *f* = 6.4 MHz. Hence, the current flowing in the RF coil dramatically increases for a given voltage excitation; this is consistent with what observed in the literature and predicted by the theoretical derivations based on Maxwell equations^30^. In particular, Fig. 6 reports the H-field maps obtained for the CAD model of Fig. 2a through full-wave simulations, without and in the presence of the metasurface at the *μ*_*r*_ = −1 point. By forcing the same circulating current in the RF coil for both the configurations, it is evident how the metasurface is able to enhance the H-field produced by the driving coil. Therefore, the herein provided circuital model is able to describe the *μ*_*r*_ = −1 condition only through the retrieved lumped parameters (13); thus, the synthesis of artificial materials can be greatly simplified.

**Figure 7:**
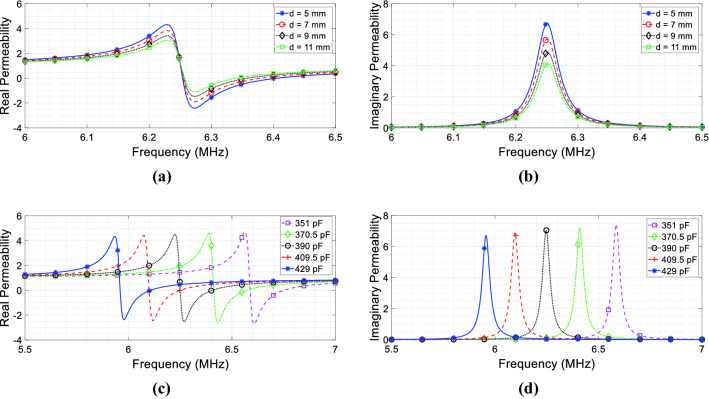
Source-related complex magnetic permeability for the CAD model of Fig. 2a, retrieved through the analytical model. (**a**, **b**) Permeability behavior when the RF coil distance from the metasurface is progressively increased; (**c**, **d**) Permeability behavior when the metasurface unit-cell capacitive load is varied. As it can be pointed out, the complex permeability experienced by the RF coil can be arbitrarily controlled.

In a practical set-up, a metasurface is fabricated by starting from a 2D array of resonant magnetic inclusions, like spiral or split ring resonators. As a matter of fact, the actual thickness of the realized metasurface is not directly correlated to the equivalent thickness of the homogeneous material adopted in full-wave simulations (Fig. 5), being extremely thinner (usually a few millimeters). As evident in (13), the retrieved permeability can be finely tailored on the basis of the lumped elements values described in our model. Hence, the metasurface has an effective thickness that can be modulated through the lumped model. In fact, the availability of a simple and straightforward circuit model, in which the relative lumped elements can be modified to shape the metasurface magnetic response according to the design requirements, is one of the major advantages of the proposed approach. In particular, we can easily adjust such parameters by noticing that *M*_12_, *L*_2_, *C*_2_ and *R*_2_ are quantities ruled by the proposed model. Therefore, by modifying the distance between metasurface and RF coil (*M*_12_), the unit-cell design (to control *R*_2_, *L*_2_ and *C*_2_) and their relative position (i.e., the array periodicity), we can obtain the desired curve for the equivalent permeability experienced by the RF coil. This implies that the proposed circuit model can also be used to characterize intermediate situations, in which, for instance, the metamaterial cannot be approximated by the semi-infinite hypothesis represented in Fig. 5. In that case, the equivalent permeability seen by the RF coil will be the average value between the air (present on one side) and an equivalent material with a diluted permeability. Several models in the literature have been developed to describe similar situations, but typically considering only the dielectric counterpart^7,39^. In this regard, Fig. 7 reports some meaningful examples of real and imaginary permeability values retrieved with the analytical model for the proposed radiating configuration. In particular, in Fig. 7a, b, the distance between the RF coil and the metasurface is varied, from 5 mm to 11 mm; this implies that the mutual coupling *M*_12_ between the RF coil and the metasurface is becoming smaller with increasing distances and, as predicted, the complex permeability amplitude related to the RF coil accordingly decreases, simulating a progressively thinner metamaterial. Additionally, in Fig. 7c, d, the analytical model is employed to retrieve the source related complex permeability when the metasurface unit-cell capacitive load is gradually changed, from 351 pF to 429 pF; as evident, the complex permeability experienced by the RF coil can be modulated and controlled, on the basis of the specific application requirements.

For instance, in^40^ it is reported that a metasurface with a pure imaginary permeability is able to perform as an ideal microwave absorber; whereas all the mathematical analysis is therein performed under the plane wave hypothesis, we can completely overcome this limit and design arbitrary metasurface complex permeabilities also considering near-field sources. This latter condition is generally closer to practical applications, especially those at relatively low operative frequency.

**Figure 8:**
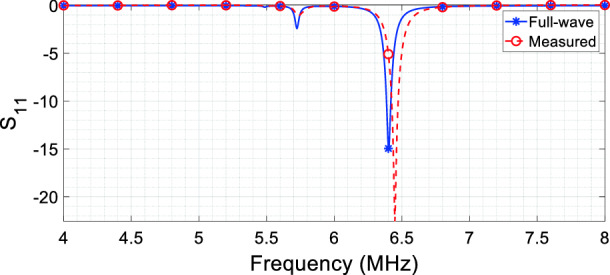
S_11_ parameter (full-wave simulation and experimental measurement) of the RF coil antenna arrangement depicted in Fig. 2a: the metasurface is able to both tune and match at 50 Ω the coil without resorting to any reactive load.

As an added value, the same metasurface can be even used to compensate any desired reactance of the coil. Before its resonant frequency, when the real permeability is positive, capacitive reactance can be compensated; conversely, after the resonant point, a negative value of the real permeability can be used to null an inductive impedance (Fig. 4a, b). Moreover, provided that the permeability imaginary component, introduced by the metasurface ohmic losses, retains the proper value to guarantee a good matching to the port impedance (12), not only the tuning of the RF coil (i.e., the cancellation of its reactive impedance component), but also the matching to the output impedance of a generator can be achieved (for instance, 50 Ω). To this aim, Fig. 8 reports both the numerical and the experimental *S*_11_ parameter of the model described in Fig. 2a. As we can see from this figure, the RF coil antenna is perfectly tuned (zero point of the real component of the effective *μ*_*r*_ in (12)) and 50 Ω-matched (proper value of the imaginary component in (12)) at 6.4 MHz by the presence of the metasurface. The resonant point of the RF coil antenna can be changed by acting on the metasurface-antenna distance (*M*_12_) or on the unit-cell design and periodicity (lumped *RLC* model), leaving to the designer degrees of freedom for the engineering of the desired solution. This can have interesting consequences in a large number of applications, as MRI.

Indeed, by a proper metasurface design, we can achieve tuning and matching of an RF coil without using any capacitive load and/or matching network. This implies a more efficient design of the RF coil, avoiding the use of lumped capacitors that are often the cause of undesirable electric field hot spots^20^.

#### Transmitter-metasurface-receiver system

By recurring to the same circuital model previously described, it is possible to express the equations system for the CAD in Fig. 2b in the following way:

14 $$\left\{ {\begin{array}{*{20}c} {Z_{11} I_{1} + Z_{12} I_{2} + Z_{13} I_{3} = V_{1} } \\ {Z_{21} I_{1} + Z_{22} I_{2} + Z_{23} I_{3} = 0} \\ {Z_{31} I_{1} + Z_{32} I_{2} + Z_{33} I_{3} = 0} \\ \end{array} } \right.$$

in which we denote with the indices 1 and 2 the fed transmitter and the passive receiver coil, respectively; in this case, the magnetic metasurface has been replaced by its equivalent resonator and addressed with index 3. We can now proceed in the same fashion as for the previous case, i.e. we express the metasurface equivalent current *I*_3_ as a function of *I*_1_ and *I*_2_ and substitute it in the first two equations of (14). The result is a 2-port system whose impedance matrix has the following form:

15 $$\left( {\begin{array}{*{20}c} {Z_{11} - \frac{{Z_{13} Z_{31} }}{{Z_{33} }}} & {Z_{12} - \frac{{Z_{13} Z_{23} }}{{Z_{33} }}} \\ {Z_{21} - \frac{{Z_{32} Z_{31} }}{{Z_{33} }}} & {Z_{22} - \frac{{Z_{23} Z_{32} }}{{Z_{33} }}} \\ \end{array} } \right)$$

**Figure 9:**
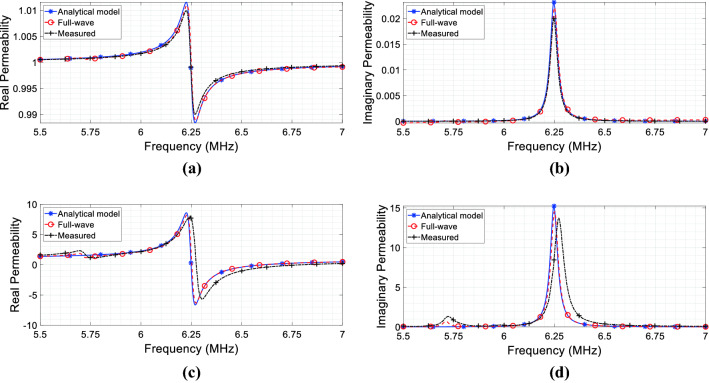
Source-related complex magnetic permeabilities for the CAD model of Fig. 2b, calculated from (17) using the lumped values versus full-wave and measured retrieved permeability. (**a**, **b**) Complex magnetic permeability $\mu_{r}^{2,2}$ experienced by the receiver. (**c**, **d**) Inductive link complex magnetic permeability $\mu_{r}^{1,2}$ between the two RF coils.

From (15), it is evident that both the transmitter and receiver self-impedances are influenced by the metasurface presence. Indeed, all the 4 terms of (15) contain a dependence on the metasurface self-impedance *Z*_33_ at the denominator, thus presenting a peak at its resonance. It is easy to verify that an expression formally equivalent to Eq. (13) can be derived for both the transmitter and receiver. Thus, by exploiting the single unit-cell design (*R*_3_, *L*_3_, *C*_3_), the cell periodicity within the array and the metasurface distance with the RF coils (*M*_13_/*M*_23_ terms), it is possible to manipulate both the reactive and the real components of the RF coils self-impedances. Following the model developed for the single coil-metasurface case, it is worth pointing out that both transmitter and receiver experience different magnetic permeabilities; hence, it immediately emerges that a magnetic metasurface acts differently on the RF coils constituting the system, depending on its relative position and on the coils geometrical constraints, as theoretically predicted. In particular, the transmitter-related complex permeability is coincident with the behavior reported in Fig. 4a, b; conversely, the receiver permeability is shown in Fig. 9a, b. It is apparent from the permeability values that the receiver is minimally affected by the metasurface presence; this is coherent with the greater distance that separates the receiver from the metasurface with respect to the transmitter (i.e., 95 mm against 5 mm).

To report a practical scenario, in resonant inductive Wireless Power Transfer (WPT), the inductive coupling is exploited to transfer energy from an active RF coil towards a passive receiving RF coil; consequently, the most important term to be studied is the off-diagonal one in (15). Indeed, the effective *Z*_11_^*eff*^ and *Z*_22_^*eff*^ (the global self-impedances of transmitter and receiver) can always be compensated by resorting to a matching network or exploiting the transmitter and receiver distances with the metasurface as an additional design parameter^15,41^. Therefore, it is worth expressing the mutual coupling term *Z*_12_^*eff*^ in its complete form to understand some interesting features on how a magnetic metasurface interacts and modifies an inductive link. Hence, we can write:

16 $$Z^{eff}_{12} = j\omega M_{12} + \frac{{\omega^{2} M_{13} M_{23} }}{{R_{3} + j\omega L_{3} + {\raise0.7ex\hbox{$1$} \!\mathord{\left/ {\vphantom {1 {j\omega C_{3} }}}\right.\kern-\nulldelimiterspace} \!\lower0.7ex\hbox{${j\omega C_{3} }$}}}}$$

where *jωM*_12_ is the classical inductive mutual coupling term between the two RF coils (*Z*_12_), in this case the transmitter and the receiver. Instead, the other additional term arises because of the metasurface presence, which is described through its equivalent resonator. By manipulating the above expression, we can directly express the source related magnetic permeability of the inductive link as:

17 $$\left\{ {\begin{array}{*{20}c} {\mu_{r}^{^{\prime}} = 1 + \frac{{\omega^{2} M_{13} M_{23} {\raise0.7ex\hbox{${M_{12} }$} \!\mathord{\left/ {\vphantom {{M_{12} } {C_{3} }}}\right.\kern-\nulldelimiterspace} \!\lower0.7ex\hbox{${C_{3} }$}} - \omega^{4} M_{13} M_{23} M_{12} L_{3} }}{{\omega^{2} M_{12}^{2} R_{3}^{2} + \left( {{\raise0.7ex\hbox{${M_{12} }$} \!\mathord{\left/ {\vphantom {{M_{12} } {C_{3} }}}\right.\kern-\nulldelimiterspace} \!\lower0.7ex\hbox{${C_{3} }$}} - \omega^{2} M_{12} L_{3} } \right)^{2} }}} \\ {\mu_{r}^{^{\prime\prime}} = \frac{{\omega^{3} M_{13} M_{23} M_{12} R_{3} }}{{\omega^{2} M_{12}^{2} R_{3}^{2} + \left( {{\raise0.7ex\hbox{${M_{12} }$} \!\mathord{\left/ {\vphantom {{M_{12} } {C_{3} }}}\right.\kern-\nulldelimiterspace} \!\lower0.7ex\hbox{${C_{3} }$}} - \omega^{2} M_{12} L_{3} } \right)^{2} }}} \\ \end{array} } \right.$$

where we have assumed that the total mutual coupling between transmitter and receiver can be expressed as:

18 $$Z^{eff}_{12} = j\omega \left( {\mu_{r}^{^{\prime}} - j\mu_{r}^{^{\prime\prime}} } \right)M_{12}$$

In Fig. 9c, d we reported the real and imaginary component of this permeability, comparing the pure analytical solution against full wave simulations and experimental measurements, obtained from the set-up depicted in Fig. 2b. Again, we observe an excellent agreement among analytical model, full-wave simulations and measurements, thus demonstrating the accuracy of the equivalent circuit in effectively representing the real scenario.

At this point, from the graphs of Fig. 9c, d, some important observations can be drawn. We immediately reveal that the metasurface is able to eliminate, almost perfectly, the mutual coupling *jωM*_12_ between transmitter and receiver. This happens slightly beyond the metasurface resonant point at *f* = 6.6 MHz (zero point cross of the real part of the retrieved permeability).

If the loss component of the retrieved permeability is low, then the metasurface acts as a perfect magnetic shield between the RF coils; indeed, the off-diagonal terms in (15) are nulled and the transmitter and receiver are decoupled. This effect has been observed in the literature and already exploited in different technological areas, as an alternative solution to ferrite shields for low frequency magnetic fields or in MRI array elements decoupling^20,23^. Obviously, this operative point must be avoided if the application under study is the wireless energy transfer between the two RF coils.

On the other hand, in WPT applications, the best working frequency results to be at the metasurface self-resonance (*f* = 6.25 MHz in Fig. 9c, d), when the reactive component of *Z*_33_ is nulled^19^ and the off-diagonal term *Z*_12_^*eff*^ is maximized. Indeed, in this configuration, the magnetic metasurface is acting as the intermediate coil of a classical 3-coil system^42^. Provided that the impedances at the port 1 and 2 (transmitter and receiver) can be appropriately compensated and matched, this operative point can lead to the maximum coupling between the two RF coils. Since efficiency is directly dependent on the square of the absolute *Z*_12_^*eff*^ value^19^, this means reaching the maximum energy delivery.

**Figure 10:**
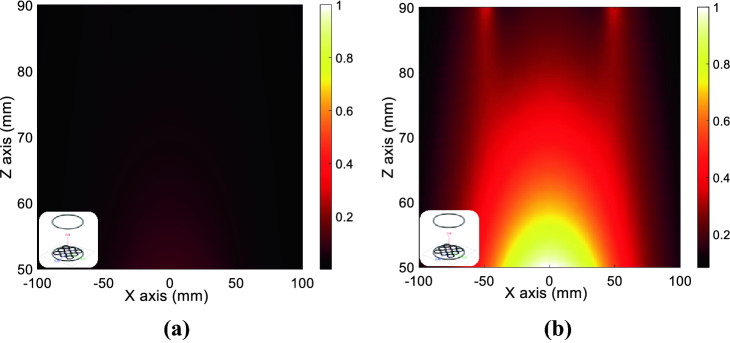
Numerical magnetic field maps evaluated for the configuration shown in the inset on a plane perpendicular to the metasurface (*xz* plane in Fig. 2b), in the space between transmitting and receiving coils. (**a**) Magnetic field distribution evaluated with the metasurface used as a magnetic field shield, at 6.6 MHz. (**b**) Same field distribution with the metasurface tuned to enhance the mutual coupling between transmitter and receiver, at 6.25 MHz. It must be noted that the comparison is performed with the same circulating current in the transmitter.

These two practically interesting working conditions, i.e. shielding and power transfer configurations, have been also evaluated through full-wave simulations. In particular, Fig. 10a reports the magnetic field distribution on the *xz* plane (refer to Fig. 2b for the geometrical reference system) between transmitting and receiving coils when the metasurface is employed as a magnetic field shield (at 6.6 MHz). Conversely, Fig. 10b describes the same geometrical configuration but with the metasurface tuned to enhance the mutual coupling between transmitter and receiver (at 6.25 MHz). It must be noticed that both these numerical experiments have been carried out for the same circulating current in the transmitting coil, to obtain a fair comparison. The obtained numerical results confirmed what theoretically expected in terms of field distribution.

Certainly, the doubt that fabricating a magnetic metasurface can be more problematic with respect to a simple additional repeater coil can be raised. However, some peculiar characteristics of magnetic metasurfaces cannot be achieved by a single additional coil, like enhanced misalignment robustness^37^ and electric field shielding^43^.

**Figure 11:**
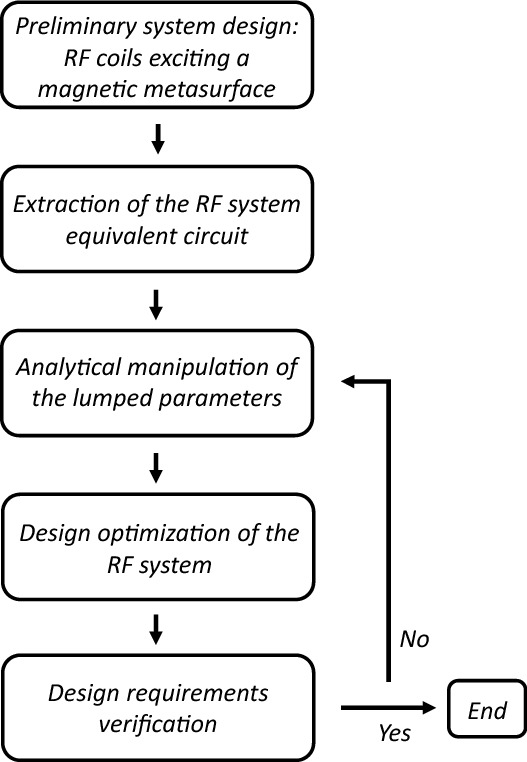
Design flowchart using the proposed equivalent circuit to facilitate the metasurface engineering step.

In conclusion, when a magnetic metasurface interacts with RF coils, understanding that each coil experiences a peculiar equivalent permeability, depending on its position and design geometries, is crucial. In this way, the various RF coils behaviors can be more easily manipulated, rather than retrieving the bulk magnetic properties of the metasurface itself, which is not convenient to describe near-field interactions. By expressing such interactions with an equivalent circuit, a straightforward and more effective design process can be accomplished, significantly aiding the engineering step, as summarized in the flow-chart scheme reported in Fig. 11.

## Discussion

In this paper, we presented a general equivalent-circuit interpretation of finite magnetic metasurfaces interacting with an arbitrary arrangement of RF coils operating in near-field regime. In particular, the developed model is able to provide a useful physical understanding, for which the metasurface complex magnetic permeability can be appropriately engineered in dependence of the various RF coils constituting the overall system. It is worth mentioning that arbitrary RF coils arrangements interacting with the metasurface can be described and analyzed, hence making the model general and easily extendible for several different applications.

We first recalled how to reduce similar structures interacting with RF coils to their own equivalent resonator model, further analyzing how a magnetic metasurface affects differently the surrounding RF coils, defining a proper source-related complex relative magnetic permeability matrix. Afterwards, we deeply studied two meaningful test-cases to validate the proposed circuital model. Firstly, we faced the single coil-metasurface system, which is the simplest possible configuration but extremely interesting for the related practical implications; secondly, we studied the classical transmitter-metasurface-receiver set-up, typical of Wireless Power Transfer applications. We compared the analytical predictions with full-wave simulations, obtaining excellent results and, thus, demonstrating the reliability and accuracy of the circuit interpretation. Moreover, measurements performed over the fabricated prototypes reinforced the numerical conclusions.

Although very detailed theoretical works describing such structures through full Maxwell equations are already available in the literature, a lumped elements model can be extremely useful in practical design and engineering process. Indeed, the possibility to quantify and manipulate the key parameters of a system results in a major advantage from a design point of view in a large number of applications, like Wireless Power Transfer and Magnetic Resonance Imaging.

The circuit model herein presented is general and we foresee an extension to electric near-field interactions between generic antennas and metasurfaces configurations.
